# Printing Parameter Requirements for 3D Printable Geopolymer Materials Prepared from Industrial Side Streams

**DOI:** 10.3390/ma14164758

**Published:** 2021-08-23

**Authors:** Qaisar Munir, Riku Peltonen, Timo Kärki

**Affiliations:** Fiber Composite Laboratory, LUT School of Energy Systems, Lappeenranta-Lahti University of Technology, 53850 Lappeenranta, Finland; riku.peltonen@lut.fi (R.P.); Timo.Karki@lut.fi (T.K.)

**Keywords:** geopolymers, 3D printing, printing parameters, recyclable materials, industrial side streams, compression, flexural strength

## Abstract

The objective of this investigation is to study the printing parameter requirements for sustainable 3D printable geopolymer materials. Side streams of the paper, mining, and construction industries were applied as geopolymer raw materials. The effect of printing parameters in terms of buildability, mixability, extrudability, curing, Al-to-Si ratio, and waste materials utilisation on the fresh and hardened state of the materials was studied. The material performance of a fresh geopolymer was measured using setting time and shape stability tests. Standardised test techniques were applied in the testing of the hardened material properties of compressive and flexural strength. The majority of developed suitable 3D printable geopolymers comprised 56–58% recycled material. Heating was used to improve the buildability and setting of the material significantly. A reactive recyclable material content of greater than 20% caused the strength and material workability to decrease. A curing time of 7–28 days increased the compressive strength but decreased the flexural strength. The layers in the test samples exhibited decreased and increased strength, respectively, in compressive and flexural strength tests. Geopolymer development was found to be a compromise between different strength values and recyclable material contents. By focusing on specialised and complex-shape products, 3D printing of geopolymers can compete with traditional manufacturing in limited markets.

## 1. Introduction

In recent years, 3D printing and additive manufacturing (AM) of concrete have gained considerable recognition because they enable the fabrication of structures from digital models [1,2]. The use of 3D printing techniques in the construction industry has the potential to increase automation [3], minimise material waste [4], and reduce labour requirements [5,6]. Moreover, 3D printing of construction materials may dramatically change conventional construction methods by permitting the adoption of advanced digital modelling practices in structural design and construction [7,8]. Among the numerous 3D printing techniques for construction products, the most familiar concrete AM process is based on material extrusion, wherein the required cementitious materials are deposited in a layering process [9,10,11]. The mixing ratios of the essential ingredients of 3D printable cementitious materials and the SiO_2_ and Al_2_O_3_ percentages of their precursors have been revealed in numerous recent publications [12,13,14,15].

The adoption of concrete 3D printing for constructing structural and architectural components has been broadly examined in present-day research [10,16,17]. Numerous techniques and materials have been investigated to promote concrete 3D printing [18,19,20]. However, various challenges involving the printing parameters, material composition, structural design, and usage of conventional materials (such as ordinary Portland cement (OPC) in terms of energy required, cost, and CO_2_ emissions are linked with the substantial limitations of the technology.

From the perspective of climate change and sustainable development, the use of OPC, which is the main constituent of concrete, can be considered problematic, as OPC production comprises nearly 5% of industrial energy consumption and is responsible for 7% of global CO_2_ emissions [21]. The production of one ton of OPC releases an average of 0.95 tons of CO_2_, primarily owing to the energy demand of the calcination of calcium carbonate. Furthermore, OPC exhibits low fire and thermal resistance and moderate chemical shielding against salts and acids [22]. Consequently, to reduce raw material usage, minimise the carbon footprint of construction activities, and improve construction quality, alternative construction materials to OPC are required. One such substitute for OPC is geopolymer cement [23].

Geopolymers have significant acid resistance [24,25], adequate adhesion to steel, concrete, and iron [26], and are considered a viable and eco-friendly substitute for OPC [7]. Geopolymer materials are created by polymerising aluminosilicate materials, activated using an alkaline solution [27]. Alkaline liquids and source materials rich in aluminium (Al) and silicon (Si) are the two essential constituents of geopolymers. Additives (glass or steel fibres) are used in geopolymers to improve their strength properties, and thixotropic additives are used to improve the printability of the geopolymer. Source materials, such as slag, fly ash, rice husk ash, silica fume, and metakaolin, among other alternatives, can be adopted for the synthesis of geopolymers [28]. The use of common precursors for geopolymers and activators allows the use of various geopolymer mixtures and mixture designs [14].

Construction and demolition (C&D) waste, which comprises inert (concrete, rock, wood, sand, bricks, etc.) and non-inert (bamboo, timber, packaging waste, etc.) materials, is a significant contributor to the overall waste volume [29]. The C&D waste generated in Europe comprises nearly 450–500 million tons every year and is considered one of the dominant sources of global waste [30]. Three types of construction sites generate C&D waste: demolition (57%), renovation (27%), and construction (16%) sites. These sites generate waste of various characteristics and compositions [31]. The essential components of C&D waste, such as bricks, concrete, wood, metals, and ceramics, have significant potential for material restoration. Therefore, the European Union has set an impressive material recovery objective (70% of generated C&D waste) in terms of recycling, recovering, or reusing such waste as construction material [32].

The recycling of C&D waste has been the subject of much research. Duan et al. [33] found that the utilisation of recycled aggregates and recycled aggregate concrete is an effective measure for addressing the growing problem of C&D waste generation and disposal. Furthermore, the use of recycled aggregates could ease the pressure on natural resources. According to Letelier et al. [34], a mix of 30% recycled aggregates and waste brick powder can be used to replace 15% of the cement used in construction without substantial losses in the strength of the final material as compared to the control concrete. Ortega et al. [35] and Xiao et al. [36] reported that the durability of concrete can be improved by using concrete brick powder. Duan et al. [33] investigated the use of mortar comprising 30% recycled powder of various C&D wastes and found that it realized flexural and compressive strengths of 89.47% and 84.43%, respectively, of cement mortar. However, mortar containing recycled powder of fine and coarse aggregates can have poor workability, which has a negative effect on the compaction properties and homogeneity of fresh concrete. Workability is an essential parameter of 3D printable geopolymer materials in addition to the particle size, mix ratio, and fresh and hardened properties.

The factors affecting the geopolymer printing process can be divided into three main categories: the geometry and size of the components, material parameters, and machine parameters. The most important material parameters are the open time and properties that affect pumping and buildability. The machine parameters (pressure, speed, and layer height) must be set according to the material properties and geometry of the component. The material must be selected based on the component to be 3D printed [37]. Geopolymer materials and 3D printing systems suitable for geopolymer printing are still under development, as illustrated by the approaches investigated in the studies of [23,37,38,39]. The AM of geopolymer materials is highly dependent on the material mixture and parameters used during extrusion. One of the major issues in the implementation of geopolymer 3D concrete printing is the development of a clear understanding of how the printing parameters are defined, such that the material can be sufficiently fluid to flow through a hosepipe without causing clogging [39].

Objects printed on a smaller scale can have similar strength properties compared to cast parts. According to Lechner et al. [40], finite element method optimisation is preferable for testing the mechanical properties of cast objects, which is state-of-the-art for various materials used on industrial scale productions. Similarly, during the casting process, the evolving thermal fields can be detected through casting process simulation [41]. However, standardized testing methods for 3D printable geopolymers or concrete are currently not available. A lack of testing guidelines makes it harder to compare and model the mechanical properties of the 3D printed elements. There is no agreement on the size of the printed object for testing and objectively characterizing the mechanical properties. The layer or interface minimal amount for testing is yet to be clarified [42].

The aim of this research was to investigate 3D printable geopolymer material parameters, such as the setting time, open time, shape stability (buildability), compressive, flexural, and bond strength, and the effect of different contents of aluminosilicate on the parameters of the geopolymer material. Geopolymers formulated from locally sourced ingredients and waste materials containing aluminium and silicon were investigated experimentally. This work is focused on the setting time, buildability, and mechanical properties, which are examined via flexural and compression testing. The effect of heat on the setting time and buildability properties of geopolymers is the focus of interest. In this study, the examination of the fresh material properties is focused on the properties of the material immediately after extrusion until when it hardens beyond extrudability. The material behaviour is studied from a 3D-printing perspective.

## 2. Experimental Preparation

### 2.1. Raw Materials

Industrial side streams and C&D waste were the primary source materials used in this study. To test the printing parameters (including setting test, shape retention, and hardened property testing) of 3D printable geopolymers, additional materials, such as bark boiler ash, mine tailings, and metakaolin, were used. Bark ash, which is a by-product of the paper and pulp industry, is used as the primary binder ingredient instead of fly ash. The differences in the Al and Si ratios in the fly ash (2) and bark boiler ash (0.9–1) were fixed using C&D waste and metakaolin. The C&D waste was employed as a filler and reactive material in this investigation because of the Al and Si ratio (between 2.8% and 4.3%). The C&D waste applied in this research was selected to have a maximum particle size of 4 mm, with larger particles of rock, soil, and Styrofoam. The Al and Si contents of the materials, which appeared as oxides, are listed in Table 1. A commercial sodium silicate solution with a molar proportion of 2.4–2.6 was used as a reagent in this study.

Sand (coarse and fine) was received as the leftover product from mine tailings and used as a filler material with no reactive components. Commercial metakaolin (having 47% of Al and Si in a 1:2 ratio) was used as a reactive pozzolan and source of Al and Si. The other materials used as additives in the geopolymer formulation were glass wool and carbon fibre. Recycled carbon fibre (chopped to 3-mm length) was used to analyse their effects on the strength properties of the 3D printable material. Bark boiler ash, C&D waste, and metakaolin were used as the binder, which is in contrast to general 3D printable mixes, wherein the binder consists of fly ash, slag, and silica fume. The moisture contents of the raw materials are listed in Table 2. The materials were placed in tightly sealed containers to prevent changes in their moisture content during the geopolymer material preparation.

### 2.2. Preparation of 3D Printable Geopolymer Materials: Mixing Procedure

**Figure 1:**
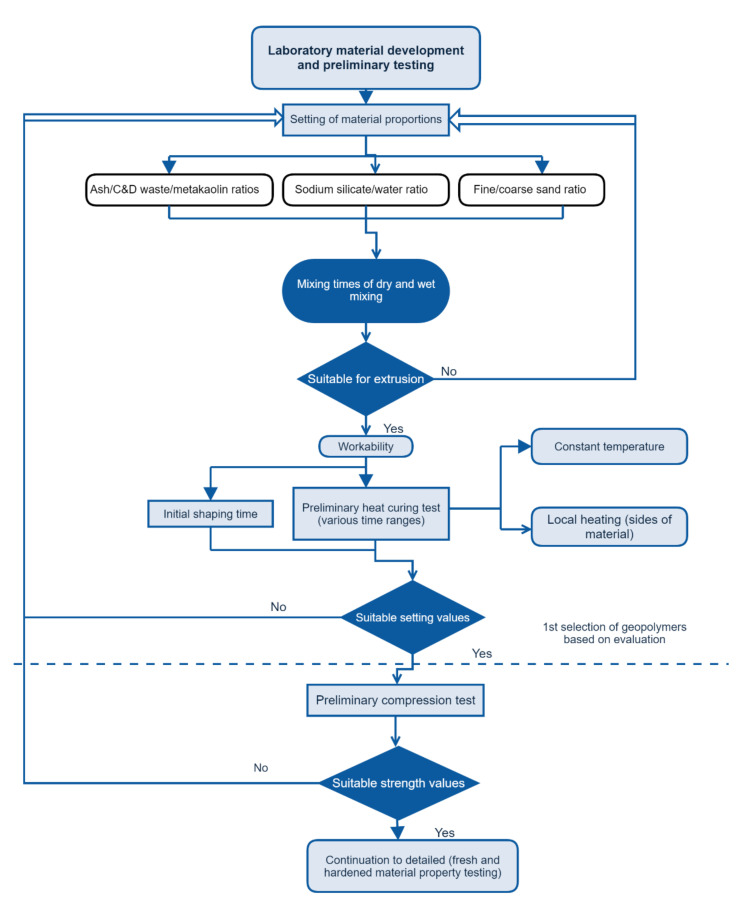
Process flow chart of laboratory experiment for material testing of geopolymer for 3D printing.

The suitable formulation of the mixture and the effect of individual components on the fresh and hardened states of the material were the criteria adopted in this study for the 3D printable geopolymer material design. In the initial development of the materials, the shaping time of the mixture was determined to obtain preliminary values to determine the possible open time of the material. Suitable mix ratios of the raw materials were selected according to the material proportions. The framework, process flow of material development, and preliminary testing are presented in Figure 1. The material testing procedure was based on flow charts presented by Panda and Tan [7] and Ma et al. [43]. The information of reported mixtures and proportions used in successful 3D printing in the literature is recognised as a starting point for laboratory testing in the development of a new 3D printable mixture [44].

The raw material mixture was prepared according to the aforementioned material proportions to achieve Al/Si ratios of 1.3–2.5. For the alkaline reagent, a sodium silicate solution having a molar ratio of 2.4–2.6 was used (31% SiO_2_ and 13% Na_2_O). The constraint applied was that no material composition could be zero, and the mixture was required to be sufficiently workable by hand before being poured into the moulds. The material ratios were adjusted according to the mixture behaviour, and the process of the mixing and determination of suitable mixing proportions evolved until an initial workability was established. The material mixture was tested for setting, curing, and extrusion before more detailed buildability and hardened property testing was conducted. The numerical values used for evaluating and selecting the geopolymer material mixtures for further testing and the parameters selected for the suitable material composition are listed in Table 3. A binder mixture containing 30–33% bark boiler ash, C&D waste, and metakaolin was selected from a series of mixtures as a starting point for the preliminary compression testing. The geopolymer material was prepared by weighing all the required ingredients with an accuracy of 0.1 g. Ash, C&D waste, metakaolin, and sand (and glass wool or carbon fibre) were mixed for 3 min. Water and sodium silicate were mixed for 1 min. After constant mixing for 1–2 min, the solution of sodium silicate and water was poured into the dry material mix. The material stuck on the sides of the mixing bowl was scraped off periodically during the mixing process to obtain an even mixture. The Hobart mixer, having a range of speeds (400 m⁻^1^), and a drill (Makita DHP453) with a mixer attachment were used for the mixing process.

The material proportion in the geopolymer mixture was established based on the initial workability of the materials. The effect of each individual component on the behaviour of the mixture was evaluated based on its total percentage in the mixture, and a decision was made regarding the addition or subtraction of an amount of material. The initial shaping times were determined to evaluate the possible open time of the materials. Heat experiments were conducted at this stage to determine possible changes in the material behaviour in terms of setting time, flow, and open time.

The material mixture was heated from the top and bottom using an air blower and heated plates at temperatures of 60–100 °C. Testing was performed only for materials with shaping times of over 5 min. The Steinel HL 2010 E heater was used, which provided the ability to vary the temperature in the range of 50–620 °C. The materials were heated between 10 and 30 s to determine whether a crust or skin would develop on the material surface. Ten geopolymer mixtures having sufficient workability and fresh properties were investigated to examine their preliminary compressive strength. The material compositions of the mixtures are listed in Table 4. Specimens were formed by casting in cubes of dimensions 50 × 50 × 50 mm and cured for 24 h in an oven at 60 °C and then cured at room temperature for 48 h to attain a maximum compression strength. The material values were changed to realise a maximum compression strength, and the effect of an individual component on the behaviour of the mixture was investigated.

### 2.3. Experimental Methods

Material testing was implemented for the mixtures in the fresh (extrudable) and hardened (cured for 7 and 28 days) states. The curing time and temperature are essential consideration in geopolymerization reaction to attain maximum strength [45]. Geopolymer material testing in the hardened state was based on a study conducted by Panda et al. [46] and Paul et al. [38], where materials were cured for 7 and 28 days to calculate strength properties, which is typically considered a standard practice for curing at ambient temperature. According to Nematollahi et al. [24], room temperature curing of geopolymer concrete for 28 days attains the same mechanical properties as 24 h oven-cured sample. Similarly, 65% of the design strength of concrete is possible to achieve after 7 days of curing. Therefore, in this study, mixtures were cured for 7 and 28 days to measure hardened material properties.

**Figure 2:**
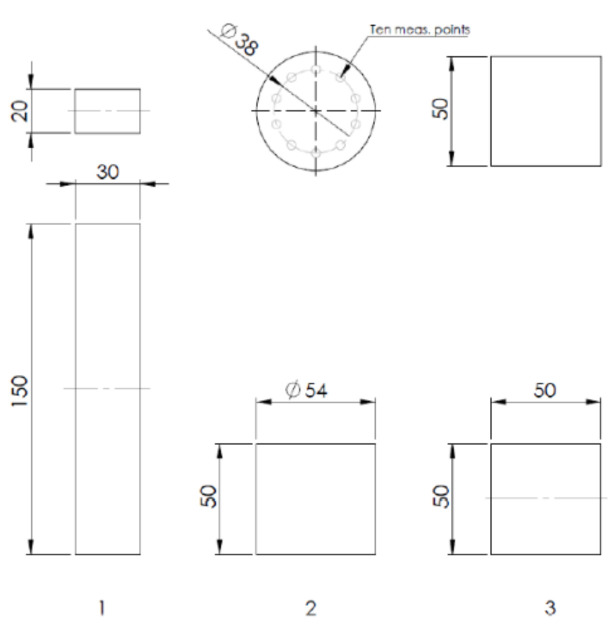
Test specimens for testing: (**1**) Flexural test, (**2**) Setting (measurement points illustrated) and shape stability test, (**3**) Compressive test.

The mixture workability was determined through the setting test, which also provided a reference for extrudability. The shape retention test provides values for the buildability of the material, which is an essential aspect in layer-upon-layer manufacturing. Compressive and flexural tests were conducted to determine the performance of the final product and to examine the hardened properties of the mixtures. Testing was performed for the two fresh materials (labelled A and B) that were evaluated and found to be most suitable for 3D printing applications after preliminary compression testing. The specimens used for testing are illustrated in Figure 2. For the testing, two different materials were prepared, and a total of 102 samples were fabricated. The distribution of the tests and the number of samples are presented in Table 5.

**Figure 3:**
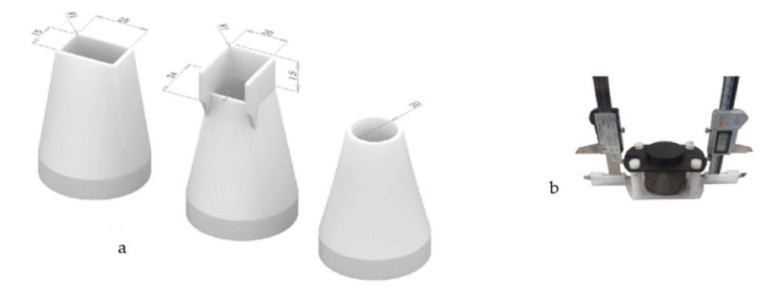
(**a**) Nozzles used in extruding with orifice dimensions, (**b**) Setup for measuring shape stability. Two digital Vernier callipers are fixed to the platform and the change in height is measured automatically.

The test techniques and the experimental methods used for 3D printable geopolymer material testing were based on study [47], as listed in Table 6. The layers for the flexural (vertical layers) and compressive testing specimens were fabricated via extrusion. The interface of the layers is indicated by the dotted line in Figure 2**.** Test specimens for testing: (1) Flexural test, (2) Setting (measurement points illustrated) and shape stability test, (3) Compressive test.. The layering was tested in two different directions using the flexural samples. The nozzles used for the layering and extrusion tests are presented in Figure 3.

#### 2.3.1. Temperature and Shape Setting

The temperature setting tests were based on a standard test for the time setting of concrete mixtures according to ASTM C403/C403M-08 [48]. The material used for testing was prepared and poured into the specimen mould. A handheld force gauge was used to perform the measurements. A round needle with a 5-mm diameter at the end of the force gauge penetrated the material perpendicularly to a depth of 25 mm at a constant rate. The measurements were obtained at 2-min intervals, and the penetration forces were recorded accordingly. Up to 13 measurements was obtained from one sample or until the penetration resistance exceeded 3.5 MPa. All the measurements were performed three times at temperatures of 20, 40, 60, and 80 °C. A heat cabinet was used for obtaining the measurements at temperatures over 20 °C. The heating cabinet used was a Gallenkamp hot box oven of size 2. A digital force gauge SAUTER FK was used for the penetration force measurements. The force gauge could be used for measurements up to 1000 N with 0.5-N accuracy.

Material buildability was measured based on shape stability by employing standard slump tests at temperatures of 20 and 100 °C. The shape stability (retention) test of the fresh material was conducted using a cylindrical specimen of diameter 54 mm and height 50 mm (thickness 2 mm). The material was poured into the cylinder and then removed after a suitable time determined by the results of the temperature setting test. The change in height was recorded using two Vernier callipers (at 0.1-mm accuracy) on opposite sides of the specimen. The setup used for the measurement is presented in Figure 3b.

#### 2.3.2. Compressive Strength Test

**Figure 4:**
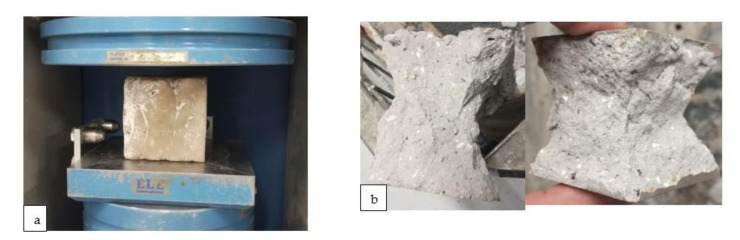
(**a**) Machine used for compression testing (**b**) sample after compression testing result.

Compressive strength testing for 3D printable materials was conducted using standard testing procedures that were developed for concrete and cement compressive strength testing. Compression tests were performed based on the standard procedure used for the hardened concrete compressive strength test SFS-EN 12390-3 [49]. The material was prepared and poured into moulds of dimensions 50 × 50 × 50 mm. Samples were prepared with room-temperature curing for 7 and 28 days. The effect of the layers on the compressive strength was determined by fabricating the specimen in two steps: the half-full mould was first extruded, and the rest of the material was then extruded after a 4-min time interval. The machine used for the compression strength tests was from ELE International, and the model was ADR Auto range with a loading rate of 0.9 kN/s used for the samples. The machine and sample used for the compression testing are presented in Figure 4a, b, respectively. 

#### 2.3.3. Flexural Strength Test

**Figure 5:**
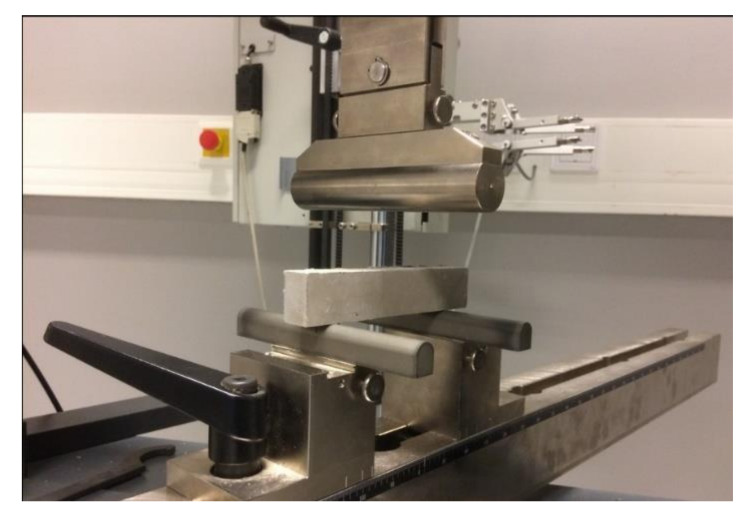
Machine used for flexural testing.

The layer behaviour of the 3D printed geopolymers and similar materials can be determined via a three-point bending test. The effect of different parameters on the flexural strength was investigated for different combinations of material composition, layer cycle time, and layer patterns. The test specimen was placed on two supports and partially between the two supports, and the third roller was above the specimen. The load was applied to the specimen by lowering the middle roller at a constant speed until the specimen fractured [29]. Flexural strength testing can be performed using standard testing methods developed for cement. The test specimens comprised 40 × 40 × 160 mm prisms [50]. Flexural testing was performed using specially fabricated specimens of 30 × 20 × 150 mm rods. Reference samples were prepared without layers by casting. Specimens for testing the material layer bond strength were fabricated by extruding the mixture to the bottom half of the mould with a syringe and a 25 × 20 mm nozzle. The specimens presented in Figure 5 were placed into a testing machine (Zwick/Roell Z020) and loaded with a centre point load at a loading rate of 3.55 N/s. The procedure was performed according to SFS-EN 12390-5 annexe A [51]. 

## 3. Results and Discussion

### 3.1. Preliminary Compression Testing

**Figure 6:**
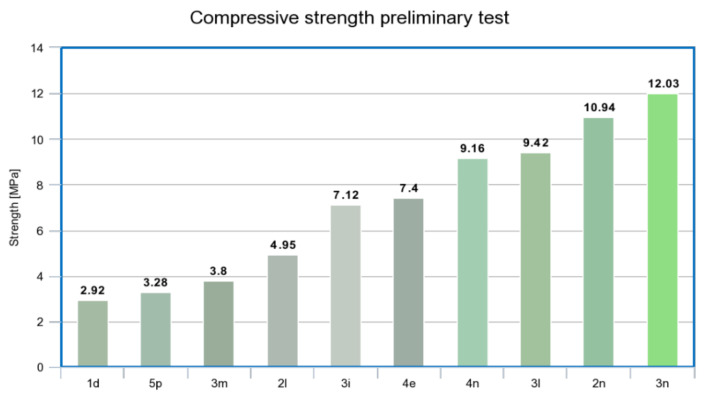
Compression values from initial compressive strength tests.

The specimens prepared for preliminary compression testing were cured at 60 °C in an oven for 24 h and then for 48 h at room temperature as presented in [47]. Figure 6 presents the compressive strength values of the mixtures from lowest to highest strength. Considering the highest compression strength results, an additional ten samples of the materials 2 n and 3 n were tested. The average compressive strength values of both materials are presented in Table 7.

Based on the compression test results, 3n was selected for the remainder of the material and 3D printability parameter testing. The second variant of 3n was formulated with a 2% addition of recycled glass wool by replacing the same quantity of metakaolin and labelled 1q. The geopolymers 3n and 1q are listed as A and B, respectively. The selected geopolymers were tested for a precise assessment of setting time, shape stability, and compressive and flexural strength.

The preliminary compression test results showed that ash alone did not proceed effectively with sodium silicate. The bark boiler ash (over 10%) exhibited a short setting time and consumed liquids before mixing with other materials, which resulted in dry and unmixed materials. A greater amount of liquid, having a 1:1 water-to-reagent ratio, is required to increase the ash quantity. The suitable performing mixture comprised 7% ash.

The results indicated that C&D waste amounts over 27% had poor strength properties and crumbled in the hand. Styrofoam spheres in the C&D waste negatively affect the strength properties and create voids and cavities in the hardened geopolymer mix. The suitable performing mixture comprised 10% C&D waste, which worked partially as an aluminosilicate source and a substitute for sand fillers. The geopolymer mix containing sand of less than 20% required high water-to-silicate ratios (greater than 1:1). The most suitable mixture comprised 39% sand, with a 3.3:2 ratio between fine and coarse sands. The moisture content in the sand caused the formation of undissolved clumps, which were excluded by drying before mixing.

A geopolymer mixture comprising 13% metakaolin was found to be the most useful, with other reactive materials at 20% or less. The mixture containing a lower volume of metakaolin exhibited weak strength properties. A water-to-reagent ratio of between 3.7 and 4.2 provided the most suitable strength and workability properties. However, the strength properties of the material decreased when the amount of water used was higher, which was also described in the study of Ma et al. [43]. A liquid volume of 28–31% provided a suitable geopolymer mixture. The addition of water to sodium silicate to decrease the viscosity and enhance workability was also presented by Panda et al. [39,52].

The addition of glass wool resulted in better workability and low adherence to the metallic surface. An additive enhancement of greater than 1% can result in clogging and separate issues in extrudability [24,44]. Carbon fibre addition results in clogging and low workability, which makes further assessment of the material unviable. The Al-to-Si ratio of the best-functioning geopolymer mixtures was approximately 1:1.5. The Al-to-Si ratio essentially influences the setting time and hardened properties.

### 3.2. Temperature Setting

**Figure 7:**
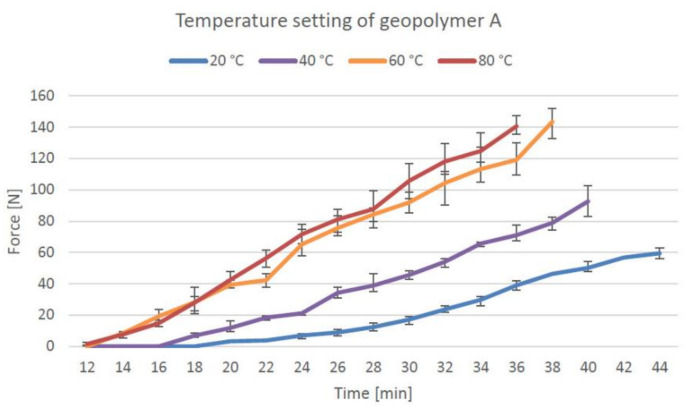
Initial setting time of geopolymer A at various temperatures.

**Figure 8:**
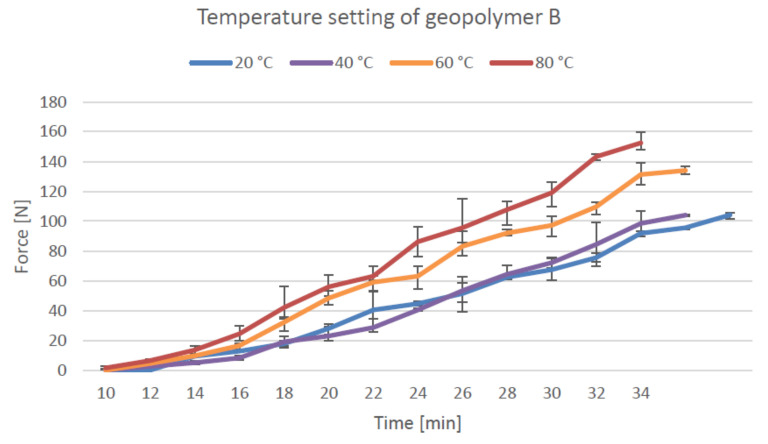
Initial setting time of geopolymer B at various temperatures.

The temperature setting test was conducted using the standard procedure for measuring the initial setting time for concrete. The curve indicates the time required for the material to reach the strength of 3.5 MPa (68.7 N), which is considered the endpoint for the initial setting time. Figure 7 presents the setting development of material A, which is presented as a line along with the measurement points. Material A reached a 60-N resistance in 44 min at 20 °C, 33 min at 40 °C, 23 min at 60 °C, and 22 min at 80 °C. The earliest point when the force was recorded from the samples was 12 min at 80 °C, and the latest value was 20 min at 20 °C. The temperature setting of geopolymer B is presented in Figure 8.

The material reaches a 60-N resistance in 28 min at 20 °C and 40 °C and in 22 min at 60 °C and 80 °C. The earliest point when the material exhibited sufficient strength for the measurement was at 10 min at 80 °C, and the latest was at 14 min at 20 °C.

The initial setting time (penetration resistance of 60 N) for geopolymer A was 50% shorter at 80 °C and 47% shorter at 60 °C than that at 20 °C, which showed that the setting time decreases at elevated temperatures. The rate of increase in setting time slows down significantly at temperatures of 60–80 °C, where the setting was only 3% faster. Both the geopolymers reached a 60-N resistance at the same time (23 min) at a temperature of 60 °C. From the time that the resistance could first be measured, geopolymer B required 18% more time to reach a 60-N resistance than geopolymer A. The glass-wool addition in geopolymer B causes the material to set faster at lower temperatures as compared to geopolymer A. The deformation drops by 61% for geopolymer A and 28% for geopolymer B when the temperature is increased from 20 to 100 °C. The application of heat for better shape stability was tested by Kazemian et al. [44] for 3D printable non-geopolymer concrete. Depending on the 3D printing application, the application of heat reduced the deformation by 71% [44]. Heat curing can shorten the curing time by 25 days if the material test is required to be performed quickly in the absence of sufficient time, for instance, a month. In 3D printing applications, the heat curing method is considered unsuitable in the majority of cases if the entire printed object must be built in a heated cabinet, which would limit the size of the objects and increase energy costs.

The geopolymerisation process is sped up at temperatures between 60 and 80 °C, and temperatures above 90 °C can have a negative impact on the mechanical properties of geopolymers. Dry-curing results in higher strengths in geopolymers than steam curing [29]. Ambient curing at room temperature usually requires 28 days of curing to achieve the same mechanical properties as 24-h oven-cured samples [24].

### 3.3. Shape Stability

**Figure 9:**
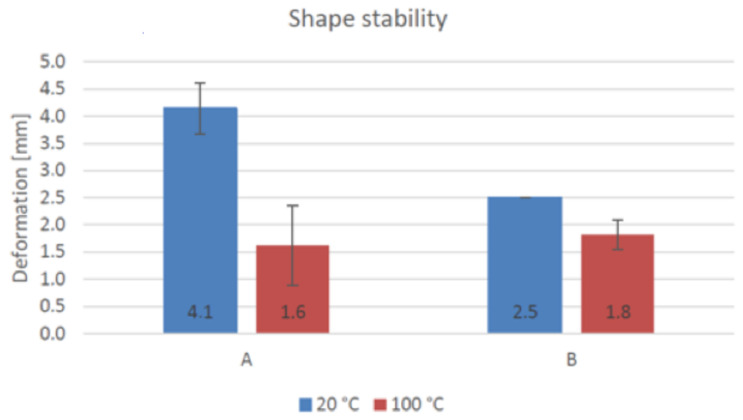
Shape stability of geopolymers A and B at 20 and 100 °C [47].

**Figure 10:**
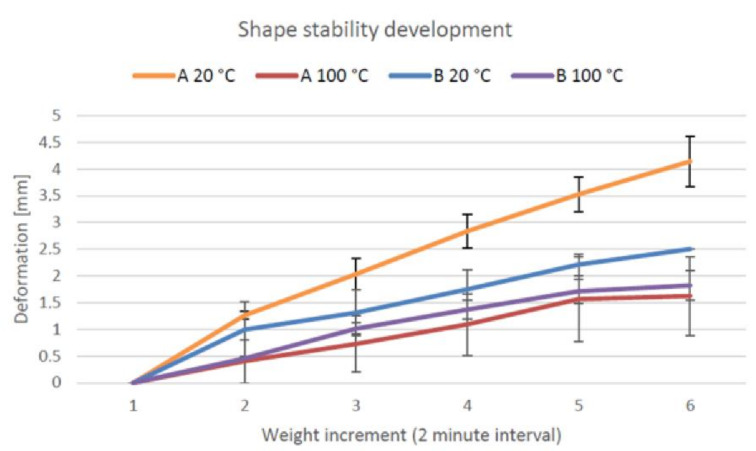
Deformation of geopolymers A and B at 20 and 100 °C [47].

The measured deformation (compression) of a 50-mm height cylinder of the material A and B samples are presented in Figure 9. The deformation for geopolymer A was 8.2% of the total height at a temperature of 20 °C and 3.2% at 100 °C. For geopolymer B, the deformations were 5% and 3.6% at 20 and 100 °C, respectively. The deformation of both geopolymers during weight addition is illustrated in Figure 10. The total weight on the geopolymers at the end of the test was 10.3 N, which equals approximately 5.3 kPa. After the final weight addition, the deformation was found to be insignificant.

Usually, the geopolymer was tested immediately after mixing, and a deformation of 2–6% was measured [29,39]. A similar deformation value was obtained in this research task after a setting time of 10 min and with the application of heat. An optimal score for a high-standard building project involved a zero-deformation rate. In terms of buildability for the suggested open and setting times after mixing, the developed geopolymers were suitable for printing with a setting time of over 10 min without the application of heat. The buildability increased as the material was allowed to set for a longer time before the extrusion. The developed materials exhibited a 20-min extrusion limit and an 8–10-min open time after mixing. In an optimal scenario, the deformation should be zero, which is required for building projects of high standard.

### 3.4. Compressive Strength Test

**Figure 11:**
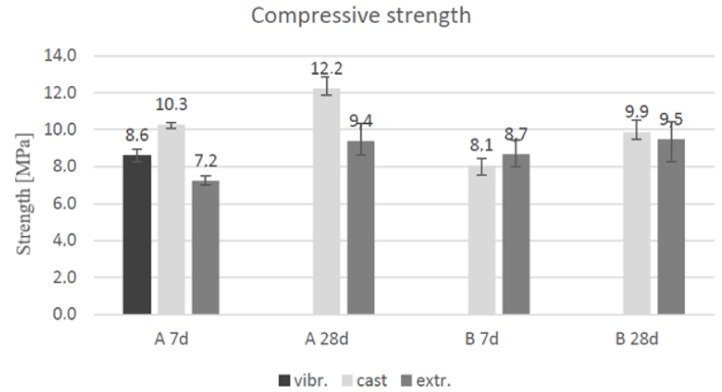
Compression strength of geopolymers A and B after 7 and 28 days of curing.

The compressive strengths of cast and layered geopolymers A and B were measured after seven and 28 days of curing at room temperature. The geopolymerisation process for various geopolymers depends on the raw materials utilisation [53]. The results of the compression tests are presented in Figure 11.

The compressive strength after curing (seven and 28 days) increased by an average of 62–63%. In the extruded specimens, for geopolymers A and B, the strength values increased by 30.6% and 9.2%, respectively. The use of fibre additives decreased the compressive strength because the fibres, or similar additives, that are parallel to the compressive loading can act as voids in the geopolymer matrix [52]. The glass-wool addition lowered the compressive strength of geopolymer B as compared to that of A, which followed the same pattern of voids in the geopolymer matrix. The results illustrated that the cast vibrated sample showed 16.5% lower strength than conventionally casted samples. According to Nematollahi et al. [24], the vibration should compact the geopolymer paste more than plain casting alone.

The density of the vibrated samples was 1.5% lower than that of the casted specimens. The porosity was apparent in every casted and extruded specimen, even after vibrating the fresh paste. This phenomenon explains the water evaporation in the chemical processes, which occurred during the geopolymerisation. All the casted and layered samples exposed during the compression testing fractured in the same manner, which is clas sified as a satisfactory failure according to SFS-EN 12390-3 (2009) [49].

### 3.5. Flexural Strength Test

**Figure 12:**
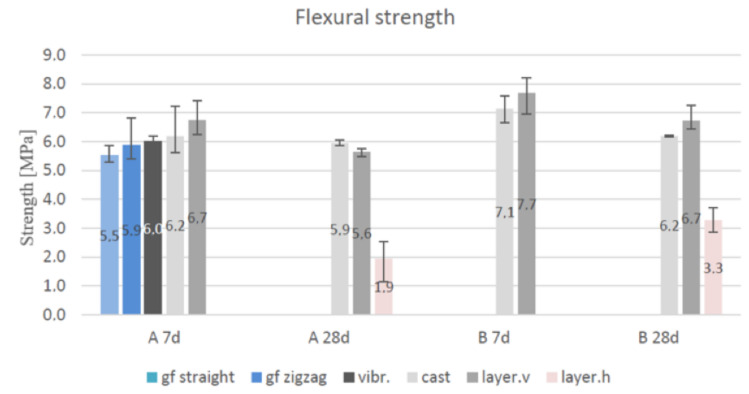
Flexural strength of specimens A and B after 7 and 28 days of curing and their variations [47].

The results of the flexural strength tests conducted for the casted and layered samples are presented in Figure 12, wherein the flexural specimens are viewed from above and the sides. All the specimens with a horizontal layer had a visible layer in the middle. The extrusion resulted in some uneven surface distributed along the length of the extrusion. A few casted specimens of both the geopolymers comprised visible defect cavities, which were observed at the bottom of the samples. Except for the aforementioned observations, all the samples exhibited an even consistency.

The strength of the specimens comprising vertical layers increased by approximately 8%, with the only exception observed in the lower strength value of the layered sample A after curing for 28 days. A decrease in strength of 13–16% was observed in the layered samples and 5–13% in the cured specimens. The porosity, lower density, and strength values of the flexural samples were identical to those of the compression samples. The vibrated test pieces exhibited a 3% and 10% decrease in flexural strength as compared to the casted specimens and vertically layered test pieces, respectively. In contrast to the compressive strength, the use of fibre additives increased the flexural strength. The increase in the flexural strength depended on the fibre length (3–6 mm or 8 mm) [24].

In this study, an increase in flexural strength of 15% was recorded between geopolymers A and B, although glass wool was used instead of fibres. The fibre addition increased the flexural strength, but also decreased the compressive strength, which could result in difficulties if both are required to be maximised.

### 3.6. Density

The compressive and flexural strengths of the specimens were measured before conducting tests for curing of seven and 28 days. The densities of the different samples are listed in Table 8. Their weight was measured in grams with a two-decimal accuracy, and the density was calculated by dividing the weight by the calculated volume. Shrinkage was considered in the density calculation as the cross-section of the specimens was measured separately. Density measurements and calculations were not performed according to the standards.

The densities of the developed geopolymers were considerably lower than that of the 3D printable geopolymers in other studies. The printed geopolymer densities were observed to be 2050 and 2250 kg/m^3^, while the densities of the casted samples were lower at 1900 and 2150 kg/m^3^, respectively [37,46]. The reported values were measured after 28 days of curing. The casted samples A and B in this study exhibited a decrease in density of 4% and 7%, respectively, from seven to 28 days. The decrease in the density of the extruded samples was 9% and 5% for the A and B geopolymers, respectively. The density of the extruded samples after 28 days of curing was 1% and 3% higher than those of the casted samples. In related studies, the density difference between the extruded and casted samples was 5–8%. Vibration unexpectedly did not result in an increased density, but instead caused a decrease in density of 3%. The average density was 25% lower in samples cured for 28 days than those in other studies. The lower density directly corresponds to the lower strength values that were observed in the property tests. The density can be higher in printed components as compared to casted components because of the pumping pressure in the extrusion phase [46].

The density of concrete differs depending on the density and quantity of the adopted aggregates, number of aggregates, entrapped air, and binder combination. The elasticity, tensile strength, and compression strength of concrete are influenced by the weight of the aggregates. Lightweight concrete is feasible for application as a filler or for insulation purposes. However, this type of concrete is unsuitable for structural purposes. The mechanical properties of concrete are significantly dependent on its density. Denser concrete typically has fewer voids and less porosity and provides greater strength. A smaller number of voids reduces the water penetration in concrete and produces stronger durability. Therefore, sufficient density and strength are required to sustain a particular loading [54].

## 4. Conclusions

This study was conducted to examine the printing parameters (mixability, initial workable time, extrudability, water-to-silicate ratio, silicate-to-binder ratio, Al-to-Si ratio, and waste materials percentage) requirements for 3D printable geopolymer materials. Specifications for 3D printable geopolymer materials in fresh (setting times, shape stability, and buildability) and hardened (compressive strength and flexural strength) states were investigated. In this study, local industrial side stream materials were used for 3D printable geopolymer formulations. The key conclusions of this study are as follows:

1. The material properties of the developed geopolymer are considered suitable for 3D printing with few prerequisites. The initial setting times of the geopolymers were investigated at approximately 38 and 44 min, which could be accelerated with the heat introduction.
2. Shape stability was observed in 8–10 min, which was enhanced by heating the geopolymer between temperatures of 20 and 100 °C.
3. The tested geopolymers cannot be extruded straight after mixing. Therefore, a controlled setting is used in a separate buffer with possible heat introduction before pumping it through an extrusion nozzle.
4. The buildability and shape stability are also increased if heat elements are attached to the nozzle with trowels.
5. Printability was significantly improved with heat treatment and suitable material proportions.
6. Decreasing the amount of recyclable materials used in the geopolymer mix increased its comprehensive strength when commercial aluminosilicate materials were substituted. Flexural strength can be enhanced with additives, which is in contrast to the case of compressive strength. The use of vibration or continuous glass fibre strings could not improve the strength properties of geopolymers, which indicates that alternative solutions are required to enhance the strength properties.
7. The development of a 3D printable geopolymer material entirely from recycled materials or waste products is challenging. The use of recycled materials in geopolymers containing aluminium/silicon alone is inadequate for material development. The ash, C&D waste, and separate side streams should be used in a suitable ratio of the elements favourable for geopolymerisation. Nevertheless, it can be concluded that recyclable products can be applied in higher volumes in 3D printable materials even as fillers.
8. The compressive strength of geopolymer concrete realised in this research work has potential applications in utility bedding, backfill walls, retaining walls or trenches, filling of sewers, tunnel-shaft construction, bedding materials for pipes, pathways, bedding for footing, pavement kerbs, patio slabs, noise barrier walls, and non-structural work. However, in the future, the aforementioned strength properties can be improved via extensive pre-processing of the recycled materials (e.g., sieving Styrofoam out of C&D waste, etc.) and by using suitable alkaline reagent ratios. The improved compressive strength material has potential for application in structural work.

## Figures and Tables

**Table 1:** Aluminium and silicon composition (weight %) of materials used in geopolymer mixture.

Component	C&D W	Bark Ash	Fly Ash	Metakaolin	Glass Wool
Al [%]	8.5	6.5	3.3	21.2	28.9
Si [%]	23.8	6.2	7.6	25.7	1.3

**Table 2:** Moisture content of the waste materials.

Material	Fly Ash	Bark Ash	Sand Fine	Sand Coarse	C&D W
Moisture [%]	10.38	0.07	11.54	2.23	21.87

**Table 3:** Parameters considered for mixture composition.

Mix	Mixability	Geopolymer Mix Initial Stage	Initial Workable Time	Extrudability	Curing Appearance After 1 Day	Water-to-Silicate Ratio	Silicate-to-Binder Ratio	Al-to-Si ratio	Waste Materials
1d	Mixed ingredients in liquid form	Sticky and low slump, partially maintains shape	Material sets under 3 min	Material started to tear after initial setting time	Cracking on surface	Above 5 (6)	Between 3.5 and 4.	Between 1.8 and 2.3	50–55%
5p	Ingredients mixed well	Sticky and low slump	More than 5 min	7.5	No powder formation/uniform colour	7.5	0	Between 1.4 and 1.6	55–60%.
3m	Ingredients mixed/liquid absorbed not well	Sticky and low slump	More than 5 min	7.5	No powder formation/uniform colour	7.5	Between 2.5 and 3	Between 1.8 and 2.3	60% or higher
2l	Ingredients mixed/liquid absorbed not well	Maintains its shape but partly sticky	More than 5 min	7.5	No powder formation/uniform colour	7.5	Between 2.5 and 3	Between 1.6 and 1.8	55–60%
3i	Ingredients mixed/ but stiffness or low viscosity	Maintains its shape but partly sticky	More than 5 min	7.5	No powder formation/uniform colour	6	Between 2.0 and 2.5	Between 1.4 and 1.6	55–60%
4e	Ingredients mixed well	Maintains its shape but partly sticky	More than 5 min	7.5	No powder formation/uniform colour	6	Between 2.0 and 2.5	Between 1.4 and 1.6	55–60%
4n	Ingredients mixed but breakdown in crumbles	Possible to shape and does not fracture	More than 5 min	7.5	No powder formation/uniform colour	7.5	Between 2.5 and 3	Between 1.4 and 1.6	55–60%
3l	Ingredients mixed/ but stiffness or low viscosity	Maintains its shape but partly sticky	More than 5 min	7.5	No powder formation/uniform colour	7.5	Between 1.5 and 2,0	Between 1.6 and 1.8	55–60%
2n	Ingredients mixed well	Sticky and low slump	More than 5 min	7.5	No powder formation/uniform colour	7.5	Between 2.5 and 3	Between 1.4 and 1.6	55–60%
3n	Ingredients mixed well	Maintains its shape but partly sticky	More than 5 min	7.5	No powder formation/uniform colour	7.5	Between 2.5 and 3	Between 1.4 and 1.6	55–60%

**Table 4:** Mixture composition (%) used for preliminary compression testing.

Mix	Ash	C&D Waste	Metakaolin	Reagent	Water	Sand c	Sand F	Al	Si	Curing Min
1 d	9	28	13	13	23		14	10.5	5.7	4
5 p	13	11		35	9	15	13	4.6	1.8	8
3 m	15	13	3	21	9	26	13	5	2.2	6
2 l	7	10	13	21	10	26	13	6.2	3.8	17
3 i	7	9	13	15	16	26	13	6.2	4	5
4 e	7	10	13	15	16	26	13	6.1	4	7
4 n	7	9.5	13	22	9	30	9	6	4	9
3 l	10	10	13	22	6	26	13	6.5	3.9	8
2 n	7	10	13	22	9	30	9	6.2	4.1	10
3 n	7	10	13	22	6	30	9	6.2	4.1	8

**Table 5:** Number of specimens for testing of materials.

Tests (Materials A and B)	Number of Samples
Compression testing (A and B)	24
Flexural testing (A and B)	24
Casted vibration specimens for compressive testing (A)	3
Flexural testing (making layers in the vertical direction for curing for 28 days) (A and B)	6
Flexural test with continuous glass fibre (A)	6
Samples casted for flexural testing in vibration table (A)	3
Setting test (A and B)	24
Shape stability (A and B)	12

**Table 6:** Experimental methods for 3D printable geopolymer material testing [47].

Test	Preparation	Specimens	Amount	Measurements
Temperature setting	Temperatures of 20, 40, 60, and 80 °C	Cylindrical container: diameter 54 mm and height 50 mm	3 samples for each temperature (12 total)	13 penetrations (2-min interval)
Shape stability	Temperatures 20 and 100 °C on sides	Cylindrical container: diameter 54 mm and height 50 mm	3 samples for each temperature (6 total)	5 layers load (2-min interval)
Compression	7- and 28-day hardened samples (cast and layered)	50 × 50 × 50 mm cube (4-min intervals for layers)	6 samples for each day (3 samples with layer structure)	Samples loaded until fracture (0.9 kN/s)
Flexural	7- and 28-day hardened samples (cast and layered)	150 × 30 × 20 mm (10-min intervals for layers)	6 samples for each day (3 samples with layer structure)	Samples loaded until fracture (3.55 N/s)

**Table 7:** Compressive strength results for the materials 2 n and 3 n.

Material Sample	Compressive Strength (MPa) (2 n)	Compressive Strength (MPa) (3 n)
1	10.93	12.02
2	10.94	12.03
3	10.93	12.02
4	10.94	12.03
5	10.92	12.03
6	10.94	12.02
7	10.92	12.01
8	10.93	12.03
9	10.93	12.02
10	10.94	12.03
Mean value	10.932	12.024

**Table 8:** Approximate density of cured geopolymers.

Sample	Sample A after 7-Days Curing [kg/m^3^]	Sample A after 28-Days Curing [kg/m^3^]	Sample B after 7-Days Curing [kg/m^3^]	Sample B after 7-Days Curing [kg/m^3^]
Casted	1693	1618	1752	1621
Extruded	1806	1635	1756	1666
Vibrated	1637	-	-	-

## Data Availability

The data presented in this study are available on request from the corresponding author.
